# Comparative evaluation of pure non-*Saccharomyces* yeasts fermentation and Lactiplantibacillus plantarum co-fermentation in fig pulp: Achieving remarkable sugar reduction and flavor enhancement

**DOI:** 10.1016/j.fochx.2025.102570

**Published:** 2025-05-20

**Authors:** Kangxue Chen, Zijian Gong, Caiyun Wu, Wenbin Rong, Ting Zhang, Qiaomei Li, Hongjie Lei

**Affiliations:** a College of Food Science and Engineering, Northwest A&F University, Yangling 712100, China; b Institute of Farm Product Storage and Processing, Xinjiang Academy of Agricultural Science, Urumqi 830091, China; c Xinjiang Lvdan Food Co., Ltd, Kashgar 844400, China

**Keywords:** Fig pulp, Non-*Saccharomyces* yeast, *Lactiplantibacillus plantarum*, Functional properties, Volatile organic compound

## Abstract

This study investigates pure fermentations of *Torulaspora delbrueckii*, *Metschnikowia pulcherrima*, and *Lachancea thermotolerans*, as well as their mixed fermentations with *Lactiplantibacillus plantarum* in fig pulp, with a focus on sugar metabolism and flavor enhancement. *T. delbrueckii* and L. *thermotolerans* demonstrated remarkable sugar reduction, decreasing glucose levels from 12.43 mg/mL to 0.34 mg/mL or lower, and fructose levels from 13.64 mg/mL to 0.08 mg/mL or lower. Mixed fermentations maintained the viability of *L. plantarum* at levels exceeding 8.16 log CFU/mL, while simultaneously enhancing the production of bioactive compounds. Analysis of volatile organic compounds (VOCs) indicated an increase in esters and terpenoids following fermentation, which contributed to the fruity, rosy, and piney aromas. Mixed fermentation exhibited superior aroma complexity compared to pure fermentation with non-*Saccharomyces* yeasts. This study provides important theoretical foundations for developing low-sugar fermented fig products with improved flavor profiles and enhanced functional properties.

## Highlights

• Comparison of fig pulp by mixed fermentations and pure fermentations was provided.
• Non*-Saccharomyces* yeasts fermentations reduced glucose and fructose contents.
• Epicatechin, rutin and ferulic acid contents increased in fermented fig pulp.
• *Metschnikowia pulcherrima* fermentation exhibited high α-amylase inhibition rate.
• Types and contents of esters and terpenoids increased after fermentation.

## Graphical abstract

**Figure:**
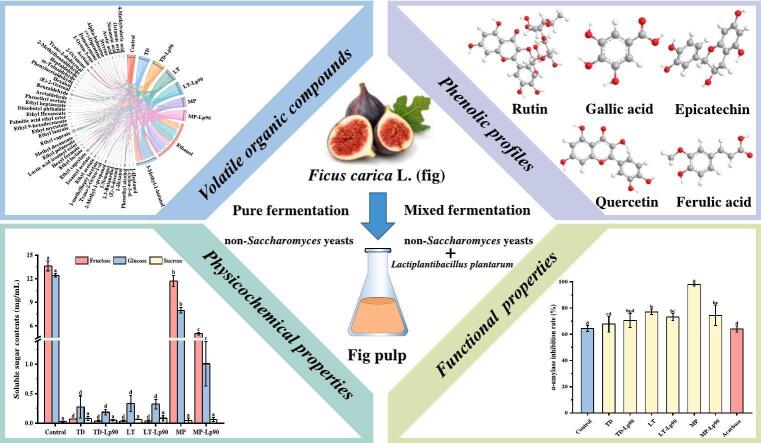

## 1 Introduction

*Ficus carica* L. (fig) is a deciduous tree or shrub belonging to the genus *Ficus* in the family *Moraceae*. Originating in the Middle East and Southwest Asia, figs are one of the oldest known cultivated fruit trees. Today, figs are cultivated extensively worldwide, with the Mediterranean region serving as the primary source of fig production. They are rich in various nutrients, including vitamins, dietary fibers, organic acids, carbohydrates, and minerals (Badgujar et al., 2014). Additionally, figs are abundant in flavonoids, carotenoids, phenolic acids, and other bioactive compounds (Sandhu et al., 2023). Figs exhibit multiple beneficial properties, such as antioxidant activities, anti-inflammatory effects, antibacterial properties, support for lowering blood sugar levels, and prevention of cardiovascular diseases (Yang et al., 2023). However, due to their high sugar content and soft texture, figs are particularly susceptible to physical damage and microbial contamination. Fresh figs can be stored for 7–8 days under low-temperature and high-humidity conditions after harvesting, while storage at 20 °C limits freshness to only 1–2 days (Mat Desa et al., 2019). Consequently, there is a pressing need for appropriate processing methods to fully utilize fig resources.

The increasing awareness of health among consumers has been propelling the growth of the worldwide functional food market in recent years. Among these, fermented fruit products have emerged as one of the most popular categories within functional foods. Non-*Saccharomyces* yeasts encompass all yeast species, excluding *Saccharomyces cerevisiae*, that are isolated from the brewing environment. Historically, non-*Saccharomyces* yeasts were regarded as undesirable or spoilage microorganisms due to their suboptimal fermentation performance, sensitivity to harsh conditions, and the production of complex aroma compounds that were not well understood (Liu et al., 2022). In recent years, an increasing number of studies have demonstrated that non-*Saccharomyces* yeast vinification can enhance wine quality by selecting appropriate inoculation protocols. This process can lead to improvements such as increased aromatic complexity, reduced alcohol content, and modulation of wine color (Chua et al., 2018). Previous research has indicated that the mixed fermentation of non-*Saccharomyces* yeasts with *Saccharomyces cerevisiae* notably enhances the glycerol, ethyl acetate, β-damascone, and terpene concentrations in icewine, thereby significantly improving its aromatic profile (Hong et al., 2021). While current research on non- *Saccharomyces* yeasts has primarily focused on enhancing wine quality, there are limited reports on their application in fig fermentation. *Lactiplantibacillus plantarum* is a prevalent species of lactic acid bacteria, extensively utilized in the production of various fermented foods, including cheese, olives, and fruit juices, owing to its exceptional adaptive and metabolic capabilities (Seddik et al., 2017). A previous study indicated that the fermentation of goji juice using *Lactiplantibacillus plantarum* significantly improved its sensory quality and enhanced its antioxidant activity, α-glucosidase inhibition capacity, and pancreatic lipase inhibition capacity (Duan et al., 2023). In addition, numerous studies have confirmed that the fermentation of *Lactiplantibacillus plantarum* is capable of producing a variety of organic acids, bioactive components, bacteriocins and volatile compounds. These metabolites positively influence the texture, nutrient content, functional properties and flavor of fermented foods (Yilmaz et al., 2022).

Therefore, the aim of this study was to investigate the effects of pure fermentations with three non-*Saccharomyces* yeasts, as well as mixed fermentations with *Lactiplantibacillus plantarum*, on the physicochemical properties, bioactive compounds, functional properties, and volatile organic compounds of fig pulp, and to provide theoretical references for the development of low-sugar functional fermented fig products.

## 2 Materials and methods

### 2.1 Activation of strains

*Lactiplantibacillus plantarum* 90 (Lp90) was provided by Shanghai Helplifes Technology Co., Ltd. (Shanghai, China). A quantity of 0.01 g of bacterial powder was incorporated into 250 mL of MRS broth and incubated at 37 °C for 14 h. Cells were collected by centrifugation (4000 ×*g*, 10 min, 4 °C) and washed twice by sterile saline before resuspended in 200 mL of sterile saline. *Torulaspora delbrueckii* (TD), *Metschnikowia pulcherrima* (MP), and *Lachancea thermotolerans* (LT) were purchased from Laffort (France). The yeasts were activated in water at 25–30 °C for 20 min before use.

### 2.2 Fermentation of fig pulp

Fresh figs were obtained from Xinjiang market (Xinjiang, China). The figs were washed and mixed with water at a ratio of 1.25:1 (*w*/*v*) for pulping. The resulting fig pulp was pasteurized at 80 °C for 20 min. Upon reaching room temperature, TD, LT, MP (0.30 g/L), and Lp90 (1 % *v*/v) were co-inoculated into fig pulp and mixed fermentations were carried out at 30 °C. Each of the three non-*Saccharomyces* yeasts was inoculated at the same initial inoculum level and subjected to pure fermentation at 20 °C.

All treatments were fermented for 72 h and samples were collected at 12 h intervals. Uninoculated pasteurized fig pulp was used as the control.

### 2.3 Determination of colony counts

Plate counting method was used to determine the colony counts. The colony counts of non-*Saccharomyces* yeasts and Lp90 were determined using YPD agar medium and MRS agar medium, respectively. Plates with 30 to 300 colonies were tallied, and the findings were presented as log CFU/mL.

### 2.4 Determination of physicochemical properties

The pH was determined utilizing a pH meter (Mettler-Toledo, Switzerland). Titratable acidity (TA) was evaluated through titration with 0.01 M NaOH, and the results were represented as percentage lactic acid. Soluble solids content (SSC) was determined using digital refractometer (Atago, Japan). Reducing sugar content was determined using 3, 5-dinitrosalicylic acid (DNS) method.

### 2.5 Determination of soluble sugar

Briefly, 0.5 g of freeze-dried fig pulp powder was combined with 10 mL of distilled water and sonicated in a water bath at 40 °C for 1 h. The resulting supernatant was obtained through centrifugation (7500 ×*g*, 15 min, 4 °C). The extract was filtered through 0.45 μm filter membranes for subsequent determination. The analysis of soluble sugars in the samples was conducted using high-performance liquid chromatography (HPLC), with an injection volume of 10 μL. The chromatographic conditions were as follows: Hypersil NH_2_ column (150 mm × 4.6 mm) was utilized with 0.02 % ammonia solution as the mobile phase at a flow rate of 1.0 mL/min. The column temperature was maintained at 35 °C, and detection was performed using the model 2414 differential refractive index detector. Glucose, fructose and sucrose in fig pulp were characterized and quantified by retention time and external standard curve.

### 2.6 Determination of total phenols, organic acids and polyphenols

The Folin-Ciocalteu method was employed to assess the total phenol content, with the results expressed in terms of gallic acid equivalents (GAE).

The organic acids and polyphenols were analyzed by HPLC. The samples were processed according to the previously reported methods with slight modifications (Veberic et al., 2008). Briefly, 1 g of lyophilized fig pulp powder was extracted with 80 % methanol solution, sonicated on an ice bath for 25 min, and the supernatant was collected by centrifugation (7000 ×*g*, 15 min, 4 °C). The extraction was conducted twice, and the supernatant was pooled and volume-determined to 10 mL. The filtered extract, obtained using 0.45 μm filter membranes, was subsequently employed for further analysis. Organic acid determination was based on previously reported methods with slight modifications (Guo et al., 2023). The mobile phases consisted of 0.01 M KH_2_PO_4_-H_3_PO_4_ (pH 2.7) and methanol in a ratio of 97:3 (*v*/v), with a flow rate of 0.6 mL/min. UV detection was performed at a wavelength of 210 nm. Polyphenols were determined according to previously reported methods (Wu et al., 2020). The mobile phases were 1 % formic acid solution (solvent A) and acetonitrile (solvent B) at a flow rate of 1 mL/min. The solvent gradient consisted of the following intervals: 0–5 min, 5 % B; 5–25 min, 12 % B; 25–40 min, 30 % B; 40–50 min, 45 % B; and 50–60 min, 5 % B. The UV detection wavelength was 280 nm. The organic acids and polyphenols in fig pulp were analyzed qualitatively and quantitatively by retention time and external standard curve.

### 2.7 Determination of antioxidant activities

#### 2.7.1 ABTS radical scavenging capacity

A mixture was prepared by combining 600 μL of diluted fig pulp with 5.4 mL of ABTS reagent. This mixture was allowed to react for 6 min in dark, after which the absorbance was recorded at a wavelength of 734 nm. The results were calculated as follows:

$$\mathrm{ABTS radical scavenging capacity}\;\left(\%\right)=\frac{A_{\text{control}}-A_{\text{sample}}}{A_{\text{control}}}\times 100$$

#### 2.7.2 Ferric reducing antioxidant power (FRAP)

A mixture was prepared by combining 0.2 mL of diluted fig pulp with 6 mL of the FRAP reagent, followed by incubated at 37 °C for 30 min. The absorbance was subsequently recorded at a wavelength of 593 nm. A calibration curve was plotted using six concentration levels of Trolox methanol solution within the range of 0–0.25 mM. The FRAP values were expressed in terms of Trolox equivalents.

### 2.8 Determination of in vitro hypoglycemic capacities

#### 2.8.1 Determination of α-amylase inhibition rate

The inhibition rate of α-amylase was assessed using a previously reported method with relevant modifications (Xia et al., 2023). Briefly, 80 μL of diluted fig pulp was mixed with an equal volume of 5 U/mL α-amylase solution and incubated at 37 °C for 10 min. Subsequently, 320 μL of 5 % starch solution was added as the substrate, mixed thoroughly, and incubated at 37 °C for 10 min. Following this, 320 μL of DNS reagent was added, and the mixture was subjected to a boiling-water bath for 5 min for appropriate dilution. The absorbance was then measured at a wavelength of 540 nm. Acarbose solution (1 mg/mL) was utilized as a positive control. The results were calculated as follows:

$$\alpha -\mathrm{amylase inhibition rate}\;\left(\%\right)=\left(1-\frac{A_{\text{sample}}-A_{\text{sample control}}}{A_{\text{blank}}-A_{\text{blank control}}}\right)\times 100$$

#### 2.8.2 Determination of α-glucosidase inhibition rate

The inhibition rate of α-glucosidase was assessed using a previously reported method with relevant modifications (Cao et al., 2018). Briefly, 2 mL of 0.2 M PBS buffer was taken to a test tube, followed by the addition of 200 μL of fig pulp and 400 μL of 0.2 U/mL α-glucosidase solution. The mixture was incubated at 37 °C for 20 min. Subsequently, 400 μL of 5 mg/mL PNPG solution was added as the substrate, followed by an additional incubation at 37 °C for 20 min. To terminate the reaction, 1.6 mL of 1 M Na_2_CO_3_ solution was added. The absorbance was recorded at a wavelength of 405 nm. Acarbose solution (0.1 mg/mL) was used as a positive control. The results were calculated as follows:

$$\alpha -\mathrm{glucosidase inhibition rate}\;\left(\%\right)=\left(1-\frac{A_{\text{sample}}-A_{\text{sample control}}}{A_{\text{blank}}-A_{\text{blank control}}}\right)\times 100$$

### 2.9 Determination of volatile organic compounds (VOCs)

#### 2.9.1 *E*-nose analysis

The aroma characteristics of fig pulp were determined using a PEN3-Plus *E*-nose (Airsense, Germany). The program parameters were configured as follows: sample pretreatment time: 5 s, cleaning time: 300 s and sample determination time: 60 s, with the carrier gas flow rate set at 400 mL/min. A volume of 2 mL of the sample was placed into a 10 mL injection bottle and allowed to equilibrate at room temperature for 160 s prior to determination.

#### 2.9.2 HS-GC-IMS analysis

A volume of 3 mL of fig pulp was placed into a 20 mL headspace injection bottle. The VOCs in fig pulp were analyzed using the HS-GC-IMS system (FlavourSpec®, G. A. S., Dortmund, Germany). The program was configured in accordance with the previously established method (Zhong et al., 2024). Qualitative analysis of VOCs was achieved by comparing retention index (RI) and drift time (DT) against the GC-IMS database and National Institute of Standards and Technology (NIST) database.

#### 2.9.3 HS-SPME-GC–MS analysis

The VOCs in fig pulp were analyzed by HS-SPME-GC–MS system with reference to the previously reported method with slight modifications (Li et al., 2021). Briefly, 5 mL of sample and 1.5 g of NaCl were added to a 20 mL headspace vial containing 10 μL of 2-octanol (0.2 mg/mL) as an internal standard, and the mixture was equilibrated for 15 min at 40 °C. Headspace micro-extraction was conducted using 75 μm Carboxen-polydimethylsiloxane (CAR-PDMS) for 30 min. VOCs were analyzed using a GC–MS system, which included a quadrupole DSQ II MS and a TR-5MS column (30 m × 0.25 mm, 0.25 μm, J&W Scientific, USA). Helium served as the carrier gas at a flow rate of 1 mL/min. The column temperature was initially maintained at 50 °C for 3 min, after which it was increased to 200 °C at a rate of 5 °C/min and held for an additional 12 min. VOCs were identified by comparing the retention times in the mass spectrograms with standard retention times in NIST database. The concentration of VOCs was analyzed semi-quantitatively using the internal standard method.

### 2.10 Statistical analysis

The data were presented as the mean ± standard deviation, derived from three independent replicated experiments. One-way ANOVA was conducted utilizing the SPSS 20 software (SPSS Inc., USA). To assess mean comparisons and identify significant differences between treatments, the Duncan test was applied (*p* < 0.05). Orthogonal partial least squares discriminant analysis (OPLS-DA) was conducted using SIMCA 14.1 software (Umetrics, Umea, Sweden), and the variable importance projections (VIP) of the predictors were calculated. Cluster heat map was generated using TBtools software (https://github.com/CJ-Chen/TBtools).

## 3 Results and discussion

### 3.1 Colony counts analysis

**Fig. 1:**
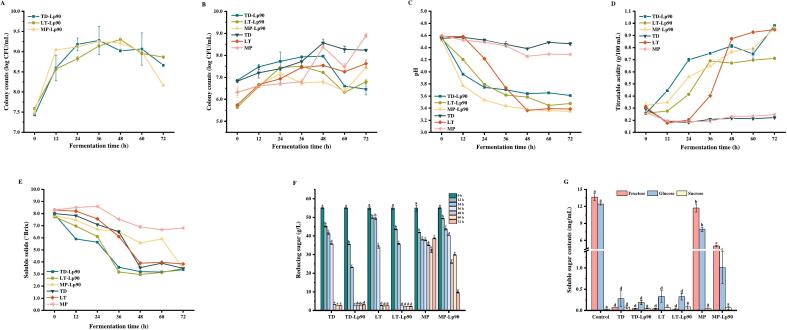
Colony counts of Lp90 (A) and non-*Saccharomyces* yeasts (B), pH (C), titratable acidity (D), soluble solids content (E), and reducing sugar content (F) during fig pulp fermentation. Changes of soluble sugar content (fructose, glucose and sucrose) in fig pulp before and after fermentation (G). The error bars indicate the standard deviation from three independent samples. Values in the same pattern with different superscript letters indicate significant differences (*p* < 0.05). Abbreviations: Control, uninoculated pasteurized fig pulp; TD-Lp90, mixed fermented fig pulp with TD and Lp90; LT-Lp90, mixed fermented fig pulp with LT and Lp90; MP-Lp90, mixed fermented fig pulp with MP and Lp90; TD, pure fermented fig pulp with TD; LT, pure fermented fig pulp with LT; MP, pure fermented fig pulp with MP.

As illustrated in Fig. 1A, no significant lag period was observed for Lp90 in fig pulp, which proliferated rapidly during the initial 12 h of fermentation. By 36 h, the highest Lp90 colony counts of 9.28 and 9.25 log CFU/mL were achieved in TD-Lp90 and MP-Lp90, respectively. At 48 h, the maximum colony count of Lp90 in LT-Lp90 reached 9.30 log CFU/mL. However, as fermentation progressed, the low pH acidic environment and elevated ethanol concentrations inhibited the growth of Lp90. Following 48 h of fermentation, the colony counts of Lp90 gradually decreased. At 72 h, the colony counts of Lp90 in all mixed fermented fig pulp remained above 8.16 log CFU/mL, which satisfies the FAO/WHO requirements for viable counts in fermented products.

As illustrated in Fig. 1B, the colony counts of non-*Saccharomyces* yeasts exhibited an increasing trend across all samples during the initial 48 h of fermentation. However, as fermentation progressed, elevated ethanol concentrations (Table S1) subsequently inhibited the growth of non-*Saccharomyces* yeasts (Gschaedler et al., 2021). Concurrently, the development of non-*Saccharomyces* yeasts was further suppressed by the biased symbiosis and antagonism observed between non-*Saccharomyces* yeasts and Lp90 in the mixed fermentation samples. Consequently, the colony counts of non-*Saccharomyces* yeasts began to decrease in all samples after 48 h of fermentation. At 72 h, the colony counts of non-*Saccharomyces* yeasts were notably higher in all pure fermentation samples. It is noteworthy that the colony counts of MP demonstrated a rising trend once again after 60 h of fermentation, peaking at 72 h, which may be attributed to the bio-protection of MP (Puyo et al., 2023).

### 3.2 Physicochemical properties analysis

The pH value serves as an indicator of the fermentation process and is crucial for evaluating the taste, acidity and organic acid content of the sample. TA exhibits a negative correlation with pH (Fig. 1C and D). During the initial 48 h of fermentation, a significant decrease in pH was observed in the fermented samples, while TA exhibited a notable increase (*p* < 0.05), with the exception of TD and MP. At 72 h, TD-Lp90 and MP-Lp90 demonstrated lower pH and higher TA compared to pure fermentation samples, indicating that Lp90 produced more acid during fermentation. In contrast, the variations in pH and TA of LT and LT-Lp90 displayed similar trends, which can be attributed to LT's ability to convert some sugars into L-lactic acid (Kapsopoulou et al., 2006).

The SSC is regarded as a compromise between the production of organic acids and the consumption of sugars throughout the fermentation process. As illustrated in Fig. 1E, the SSC of unfermented fig pulp was approximately 8°Brix. Previous research has indicated that the fermentation capacity of MP is notably weak (Morata et al., 2019). At 72 h, the SSC of MP measured 6.8°Brix, whereas all other fermentation samples recorded values below 3.84°Brix. This significant decrease in SSC suggests rapid growth and metabolism, leading to accelerated sugar consumption by the three non-*Saccharomyces* yeasts and Lp90 during fermentation. The levels of reducing sugar serve as indicators of carbon source utilization by the strains throughout the fermentation process (Fig. 1F). At 72 h, the levels of reducing sugars were significantly decreased in each sample (*p* < 0.05). In comparison to the pure fermentation samples, the mixed fermentation samples exhibited a faster rate of sugar reduction. This can be attributed to the addition of Lp90, which utilizes sugar faster for metabolism and proliferation. The changes in glucose, fructose and sucrose contents in fig pulp before and after fermentation were further analyzed (Fig. 1G). Glucose and fructose emerged as the predominant soluble sugars in fig pulp, while the sucrose content was measured at only 0.031 mg/mL. This phenomenon can be attributed to the high activity of converting enzymes during fig ripening, which irreversibly transformed sucrose into glucose and fructose (Wang et al., 2024). At 72 h, a significant decrease in glucose and fructose levels was observed across all samples (*p* < 0.05), whereas the level of sucrose remained relatively unchanged. This indicated that glucose and fructose served as the primary carbon sources for the growth of the three non-*Saccharomyces* yeasts and Lp90. Notably, MP and MP-Lp90 exhibited a higher consumption of glucose, while the other four sample groups preferentially utilized fructose. This indicates that MP was more inclined to utilize glucose, whereas TD and LT showed a greater tendency to metabolize fructose. These findings were in alignment with previous research (Chua et al., 2018). Overall, the various fermentation strains shared both common characteristics of utilizing sugars and presented their own unique properties of metabolizing sugars.

**Fig. 2:**
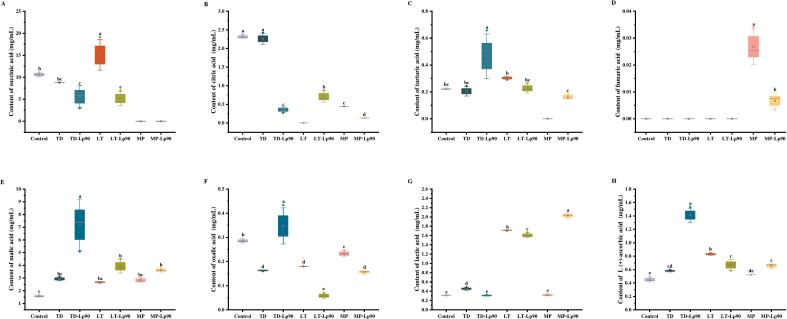
Changes of organic acid content in fig pulp before and after fermentation: succinic acid content (A), citric acid content (B), tartaric acid content (C), fumaric acid content (D), malic acid content (E), oxalic acid content (F), lactic acid content (G), and L (+)-ascorbic acid content (H). The error bars indicate the standard deviation from three independent samples. Values in the same pattern column with different superscript letters indicate significant differences (*p* < 0.05).

Fig. 2 shows the changes in organic acid contents in fig pulp before and after fermentation. Succinic acid (Fig. 2A) and citric acid (Fig. 2B) were identified as the predominant organic acids in unfermented fig pulp, with contents of 10.61 mg/mL and 2.32 mg/mL, respectively. It has been demonstrated that in yeast cells, pyruvate can react with carbon dioxide to form oxaloacetate through a carboxylation reaction. This compound subsequently participates in the tricarboxylic acid cycle and is reduced to malic acid and other intermediates (Zelle et al., 2008). After fermentations, malic acid levels increased in all samples (Fig. 2E), with TD-Lp90 exhibiting the highest concentration of malic acid at 7.20 mg/mL, reflecting a significant increase compared to unfermented fig pulp. Lactic acid contents were significantly higher in all samples except MP and TD-Lp90 (*p* < 0.05) (Fig. 2G). Lactic acid can be generated through the reduction of pyruvate and the transformation of malic acid (Cousin et al., 2017). Its milder acidic taste enhances the flavor and mouthfeel of the sample when present in appropriate amounts. Citric acid contents were significantly lower in all fermentation samples except TD (Fig. 2B). This phenomenon may be attributed to the metabolic processing of citric acid by Lp90 during fermentation, which facilitates the synthesis of additional organic acids. The previous study has demonstrated that certain non-*Saccharomyce*s yeast species are capable of degrading citric acid (Zhong et al., 2020). In this study, citric acid was significantly reduced in MP and was not detected in LT, suggesting that LT and MP may also have the ability to degrade citric acid. Additionally, L (+)-ascorbic acid is known for its various beneficial effects, including antioxidant properties, promotion of collagen synthesis, and enhancement of immune function (Zheng et al., 2022). L (+)-ascorbic acid contents were significantly elevated in all fermentation samples except for MP (*p* < 0.05) (Fig. 2H).

### 3.3 Phenolic profiles

**Fig. 3:**
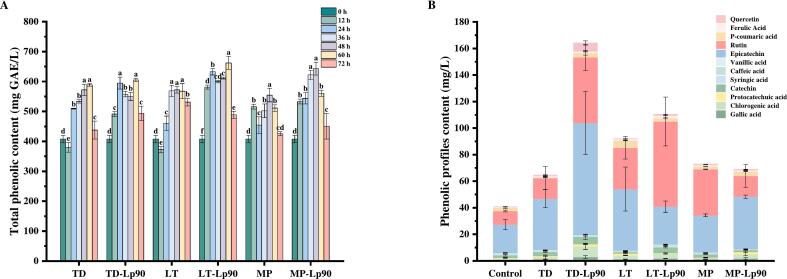
Changes in total phenolic content during fig pulp fermentation (A). Phenolic components of fig pulp before and after fermentation (B). The error bars indicate the standard deviation from three independent samples. The values marked with different superscript letters indicate significant differences within treatment groups (*p* < 0.05).

Phenolic compounds represent a crucial class of secondary metabolites found in plants, playing a vital role in protecting them from UV radiation and pathogens. Prolonged consumption of phenolic-rich foods has been associated with the prevention of cancer, diabetes, neurological disorders, cardiovascular disease, osteoporosis, and various chronic diseases (Gao et al., 2022). As illustrated in Fig. 3A, there was a significant increase in the total phenol content across all samples during the initial phase of fermentation (*p* < 0.05). LT-Lp90 exhibited the highest total phenol content, measuring 662.24 mg GAE/L. This increase can be attributed to the enzymatic breakdown of cell wall structures by microorganisms, which facilitates the release of bound phenolic compounds from plant cells (Vivek et al., 2019). However, after 60 to 72 h of fermentation, a reduction in total phenolic content was observed. This decline may be due to the binding, condensation or adsorption of phenolic compounds with various substances, such as proteins (Escudero-López et al., 2013). Nevertheless, the total phenol levels in all samples after fermentation were significantly higher compared to those in the unfermented sample.

**Fig. 4:**
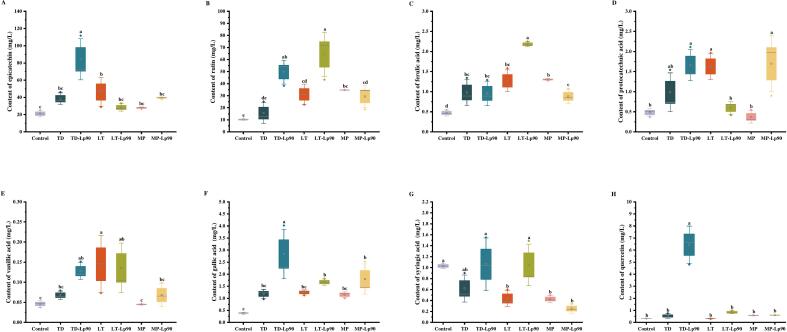

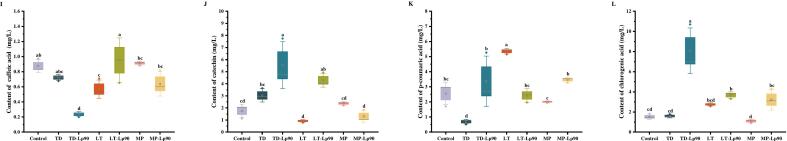
Changes of phenolic profile content in fig pulp before and after fermentation: epicatechin content (A), rutin content (B), ferulic acid content (C), protocatechuic acid content (D), vanillic acid content (E), gallic acid content (F), syringic acid content (G), quercetin content (H), caffeic acid (I), catechin content (J), and p-coumaric acid content (K). The error bars indicate the standard deviation from three independent samples. Values in the same pattern column with different superscript letters indicate significant differences (*p* < 0.05).

A total of 12 phenolic compounds were identified (Fig. 3B), with epicatechin (52 %) and rutin (25 %) being the predominant compounds in unfermented fig pulp. Both epicatechin and rutin are recognized for their potent antioxidant properties, which neutralize free radicals and mitigate cellular aging and damage. Additionally, they exhibit anti-inflammatory, cholesterol-lowering (Hao et al., 2012), immune-enhancing, and neuroprotective effects against neurodegenerative diseases (Muvhulawa et al., 2022). Following fermentation, the total concentration of phenolic compounds increased across all samples, with TD-Lp90 exhibiting the highest total concentration of 164.15 mg/L, indicating that fermentation facilitates the release of phenolic compounds. Ferulic acid, known for its superior anti-inflammatory and antibacterial properties (Zduńska et al., 2018), was found to be elevated in all samples after fermentation (Fig. 4C). Conversely, the levels of syringic acid significantly decrease in all samples except for TD, TD-Lp90 and LT-Lp90 (Fig. 4G). This reduction may be attributed to the microbial strains converting syringic acid into various metabolites, including alcohols, acids and esters during fermentation (Starowicz et al., 2024). Additionally, a notable decrease in caffeic acid levels was observed in both TD-Lp90 and LT (Fig. 4I), likely due to the action of reductase and decarboxylase enzymes present in the strains, which convert caffeic acid into ethyl catechol and vinyl catechol (Svensson et al., 2010). The p-coumaric acid content was significantly lower in TD, while LT exhibited a significantly higher concentration (Fig. 4K). It had been demonstrated that p-coumaric acid can be degraded into p-vinylphenol, however, the extent of this transformation varies considerably among different strains (Zheng et al., 2022).

### 3.4 Antioxidant activities analysis

**Fig. 5:**
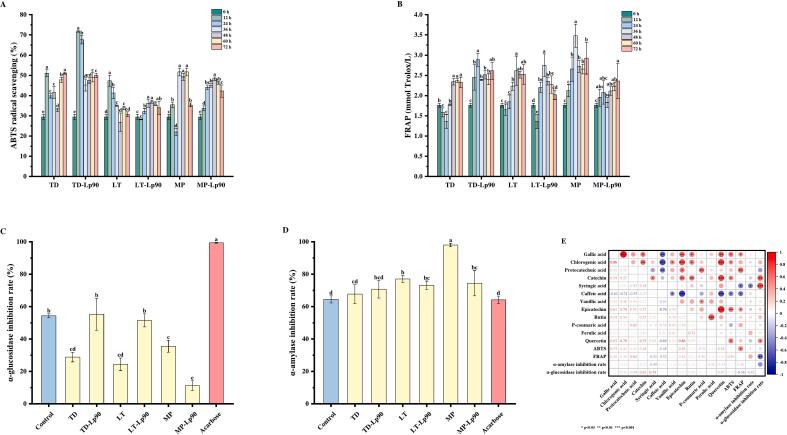
Antioxidant activities based on ABTS (A) and FRAP (B) during fig pulp fermentation. In vitro hypoglycemic activities based on the inhibition rates of α-glucosidase (C) and α-amylase (D) in fig pulp before and after fermentation. Heat map of Pearson correction coefficient between antioxidant activities, in vitro hypoglycemic activities and phytochemicals. *Correlation is significant at *p* < 0.05. **Correlation is significant at *p* < 0.01. ***Correlation is significant at *p* < 0.001. The error bars indicate the standard deviation from three independent samples. Values in the same pattern with different superscript letters indicate significant differences (*p* < 0.05).

The evaluation of antioxidant activities in fig pulp throughout the fermentation process was conducted by assessing the ABTS radical scavenging capacity (Fig. 5A) and the FRAP methods (Fig. 5B). At 72 h, there was a significant increase in the ABTS radical scavenging capacities across all groups (*p* < 0.05), with the exception of LT, which exhibited no significant change. TD demonstrated the highest ABTS radical scavenging capacity, showing an increase of 73.35 % compared to the unfermented fig pulp. Additionally, at 72 h, all samples exhibited a significant increase in FRAP (*p* < 0.05), except for LT-Lp90, which showed no significant change. MP displayed the highest FRAP, which was 66.48 % greater than that of the unfermented sample.

Pearson correlation analysis was performed to assess the relationship between antioxidant activities and polyphenol contents (Fig. 5E). The ability to scavenge ABTS radicals exhibited a significant positive correlation with the levels of quercetin, catechin, gallic acid, and chlorogenic acid (*p* < 0.05). A highly significant positive correlation was also observed with the level of epicatechin (*p* < 0.01), whereas a significant negative correlation was identified with the content of caffeic acid (*p* < 0.05). Additionally, FRAP demonstrated a significant positive correlation with the levels of both epicatechin and gallic acid (*p* < 0.05), as well as a highly significant positive correlation with the content of protocatechuic acid (*p* < 0.01). In contrast, significant negative correlations were observed with the levels of syringic acid and caffeic acid (*p* < 0.05).

### 3.5 In vitro hypoglycemic activities analysis

α-Glucosidase and α-amylase are two crucial hydrolyzing enzymes involved in carbohydrate metabolism and blood sugar regulation. Inhibiting their activities can slow the hydrolysis of complex carbohydrates in the gastrointestinal tract, thereby preventing a rapid increase in blood sugar levels following meals (Zhang et al., 2017). The inhibition rates of α-glucosidase and α-amylase in fig pulp, both before and after fermentation, were shown in Fig. 5C and D. The α-glucosidase inhibition rates were significantly reduced in all fermentation samples (*p* < 0.05), with the exception of TD-Lp90 and LT-Lp90. Notably, MP-Lp90 exhibited the lowest α-glucosidase inhibition rate at 11.21 %. Conversely, the α-amylase inhibition rates significantly increased in all fermentation samples (*p* < 0.05), except for TD and TD-Lp90, which showed no significant change. TD recorded the lowest α-amylase inhibition rate at 67.70 %, while MP exhibited the highest inhibition rate of 98 %.

The previous research has demonstrated that phenolic compounds can bind to digestive enzymes, thereby altering their activities (Mojica et al., 2015). A Pearson correlation analysis was conducted to evaluate the relationship between enzyme inhibitory capacities and polyphenols concentrations (Fig. 5E). The inhibition rates of α-glucosidase exhibited a significant positive correlation with quercetin levels (*p* < 0.05), and a highly significant positive correlation with the concentrations of syringic acid and catechin (*p* < 0.01). In contrast, the inhibition rates of α-amylase exhibited a significant negative correlation with the content of syringic acid (*p* < 0.05).

### 3.6 VOCs analysis

#### 3.6.1 *E*-nose analysis

**Fig. 6:**
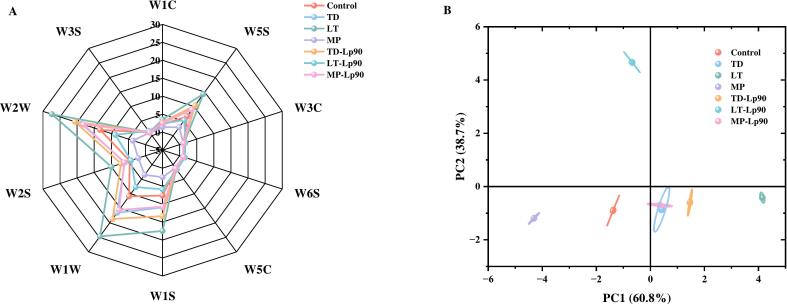
The radar chart (A) and PCA analysis (B) of VOCs in fig pulp before and after fermentation.

Changes in the overall flavor profile of fig pulp before and after fermentation can be effectively characterized using *E*-nose. The radar chart is presented in Fig. 6A. The response values for W5S, W1S, W1W, W2S, and W2W were higher than those of the other sensors across all samples, with the exception of MP. This indicates that the primary VOCs present in fermented fig pulp included nitrogen oxides, methyl derivatives, sulfides, alcohols, aldehydes, ketones, and aromatic substances. Principal component analysis was conducted (Fig. 6B), where PC1 and PC2 captured the majority of the information regarding VOCs. Notably, some regions of TD and MP-Lp90 overlapped, suggesting that these two fermented samples may exhibit similar aroma profiles. In contrast, the remaining fermentation samples can be effectively distinguished on the distribution map.

#### 3.6.2 HS-GC-IMS analysis

**Fig. 7:**
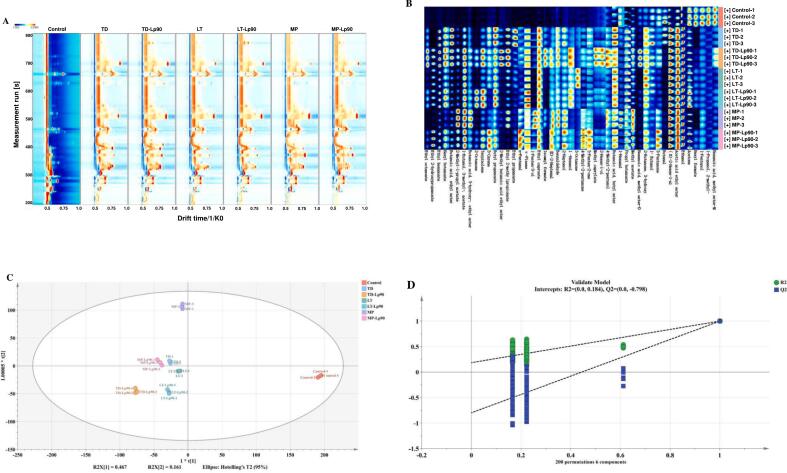

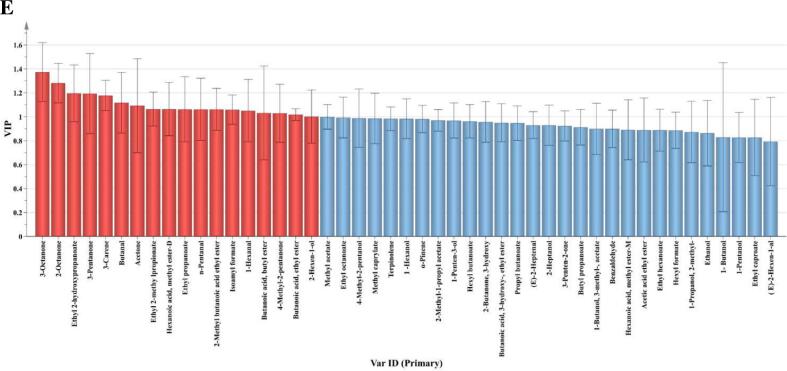
Two-dimensional chromatogram spectrum (A) and fingerprint (B) of VOCs in fig pulp before and after fermentation. OPLS-DA score plot (R^2^Y = 0.992, Q^2^ = 0.983) (C). Cross-substitution plot of 200 permutation tests (R^2^ = 0.184, Q^2^ = −0.798) (D). Distribution of VIP values (red represents the characteristic flavors with VIP > 1) (E). (For interpretation of the references to color in this figure legend, the reader is referred to the web version of this article.)

GC-IMS represents an innovative approach for flavor analysis, characterized by straightforward sample preparation, high sensitivity, exceptional accuracy, and rapid detection speed (Ma et al., 2024). This technique enables precise qualitative analysis of VOCs in fig pulp. The topographical map of VOCs was constructed, using unfermented fig pulp as a comparative reference (Fig. 7A). The majority of the signals were detected within the retention time range of 200 to 700 s and drift times between 0.5 and 1.0 s. In comparison to the control, a noticeable increase in both the variety and concentration of VOCs was observed across all fermented fig pulp. Furthermore, the distributions of VOCs, represented by the red regions on the map, varied among the treatments, with TD-Lp90 exhibiting the most abundant red regions. This suggests that VOC profiles of fermented fig pulp differ significantly based on the fermentation strains employed. Characteristic fingerprints were constructed to elucidate the variations among each volatile compound (Fig. 7B). A total of 46 VOCs were identified, consisting of 21 esters, 9 alcohols, 7 ketones, 6 aldehydes, and 3 terpenes. In unfermented fig pulp, esters and alcohols predominated, with hexyl formate and hexanoic acid, methyl ester imparting sweet fruit aromas reminiscent of apple and pineapple. Notably, the concentrations of the principal VOCs initially present in fig pulp diminished to varying degrees following fermentation. Concurrently, the fermentation process significantly enhanced both the diversity and concentration of VOCs in the fig pulp. In comparison to pure fermentations, mixed fermentations of fig pulp exhibited a greater richness in VOCs, suggesting that the addition of Lp90 facilitated the release of additional VOCs. Preliminary comparisons indicated that the peak intensities of esters, alcohols, and aldehydes associated with TD-Lp90 were elevated, particularly with an increased accumulation of esters. Many of these esters (e.g., butyl propanoate, isoamyl formate, ethyl caproate) are characterized by pleasant fruity and rosy aromas. Furthermore, three new terpenes were generated post-fermentation, contributing citrus and pine notes to the fig pulp.

The 46 VOCs identified through GC-IMS acted as the dependent variable, and different fermentation treatments were employed as independent variables for the OPLS-DA. As shown in Fig. 7C, distinct separation between the MP and Control groups was observed in quadrants 2 and 4, respectively, whereas the other fermentation samples were clustered more closely together. In this analysis, the independent variable fit index (R^2^X) was 0.977, the dependent variable fit index (R^2^Y) was 0.992, and the model prediction index (Q^2^) was 0.983, indicating an excellent model fit. In order to ensure the robustness and credibility of the model, 200 substitution tests were conducted. As illustrated in Fig. 7D, the point where the Q^2^ regression line intersects the vertical axis is below zero, suggesting that the model is not overfitted and that the validation process is successful. The results indicated that unfermented fig pulp and MP could be effectively distinguished from the other fermented samples based on their flavor profiles. To explore the contribution of various aromatic compounds to fig pulp flavor, a total of 18 differential aroma substances were identified based on the criterion of VIP exceeding 1 (Fig. 7E). These included 8 esters, 5 ketones, 3 aldehydes, 1 alcohol, and 1 terpenoid. Upon further analysis, the differential aroma substances can be categorized into two distinct groups. The first group comprises acetone, 3-pentanone, butanal, and 1-hexanal, all of which possess irritating odors and are present in high concentrations in unfermented fig pulp. The second group consists of the remaining 14 differential aroma substances, which are significantly found in fermented fig pulp. These substances exhibit pleasant sweetness and fruity characteristics, positively contributing to the enhancement of fig pulp flavor.

#### 3.6.3 HS-SPME-GC–MS analysis

**Fig. 8:**
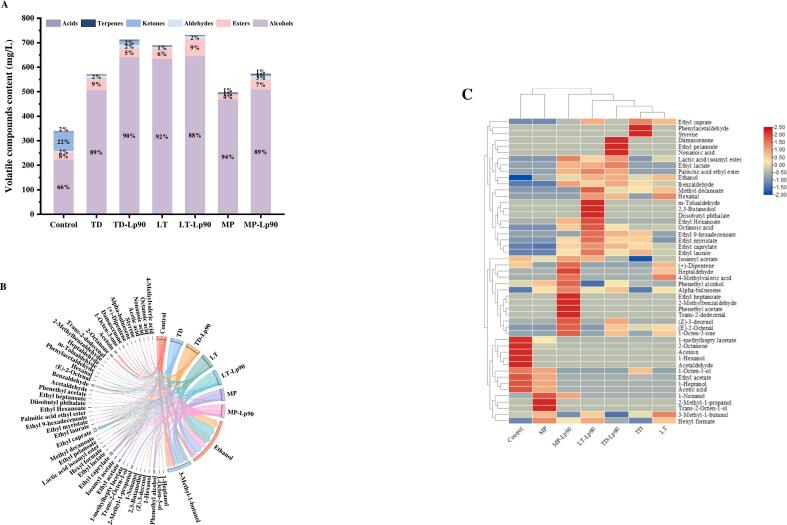
Stacked diagram (A), chord diagram (B) and heat map and hierarchical clustering (C) of VOCs in fig pulp before and after fermentation.

Although GC-IMS offers numerous advantages, including high sensitivity and excellent separation selectivity, its ability to detect ionic compounds is limited to those with a higher proton affinity than water (Gu et al., 2021). Conversely, GC–MS is a widely employed method for flavor analysis, supported by standard reference libraries that provide detailed information about the compounds under investigation (Nie et al., 2022). A total of 49 VOCs including 11 alcohols, 18 esters, 9 aldehydes, 4 ketones, 3 terpenes, and 4 acids were detected in fig pulp using GC–MS (Table. S2). As illustrated in Fig. 8A, the total concentrations of VOCs increased in all samples following fermentation, with mixed fermented fig pulp exhibiting higher levels compared to pure fermentations. This observation aligns with the findings from the GC-IMS assay. Notably, fourteen new esters were produced post-fermentation, with LT-Lp90 yielding the highest concentration of esters at 69.17 mg/L, followed by TD at 49.69 mg/L. As shown in Fig. 8B, acetic acid ethyl ester as the predominant ester compound in fig pulp, contributing a pleasant fruity aroma. The levels of ethyl octanoate were significantly elevated in all samples except for MP, imparting a sweet pineapple flavor. In unfermented fig pulp, 2-octanone and acetoin were identified as the primary ketones, characterized by a naturally woody herbal note and buttery flavor. Notably, acetoin was undetectable in all fermented samples, while the concentration of 2-octanone showed a significant decrease. Small amounts of 1-octen-3-one and damascenone were produced in TD-Lp90, LT and MP-Lp90, introducing creamy and rosy aromas to the samples. Additionally, the total terpenoid content increased across all samples after fermentation, with the generation of two new terpenoids that added pine and floral notes to the fig pulp. To further investigate the differences in VOC profiles in fig pulp fermented with various strains, a hierarchical cluster analysis was conducted (Fig. 8C). The fig pulp samples were classified into four clusters: the first cluster comprised unfermented fig pulp; the second cluster included MP; the third cluster encompassed MP-Lp90; and the remaining fermented samples constituted the fourth cluster, which was similar to the results of GC-IMS.

Overall, all three assays effectively characterized the VOCs present in fig pulp. The β-glucosidase enzymes found in non-*Saccharomyces* yeasts hydrolyze non-volatile glycosidic precursors in the raw material, resulting in increased levels of VOCs (Graf et al., 2022). In this study, the fermentation process utilizing non-*Saccharomyces* yeasts significantly enhanced in the prevalence of VOCs in fig pulp. In addition, the esterifications of certain alcohols and acids, catalyzed by the complex enzyme systems of Lp90, contributed to a richer profile of esters in the mixed fermentations.

## 4 Conclusion

Fig pulp was prepared through pure fermentation using non-*Saccharomyces* yeasts (TD, LT, and MP) and mixed fermentation with Lp90. The fermentation process resulted in an increase in phenolic compounds such as epicatechin, rutin, ferulic acid, quercetin, and gallic acid in the fig pulp, as well as enhancements in ABTS free radical scavenging, FRAP, and α-amylase inhibition. A variety of esters, such as ethyl caproate, butyl propanoate and ethyl octanoate, were generated during fermentation, contributing to the fig pulp's pleasant aroma. The overall quality of fig pulp was further improved through mixed fermentation compared to pure fermentation. Furthermore, the three non-*Saccharomyces* yeasts exhibited distinct metabolic characteristics, with TD and LT demonstrating a higher capacity for sugar metabolism and a notable reduction in reducing sugar content in the fig pulp post-fermentation, while MP displayed superior α-amylase inhibition ability. Overall, this study offers new insights for future fig processing.

## CRediT authorship contribution statement

**Kangxue Chen:** Writing – original draft, Software, Methodology, Investigation, Conceptualization. **Zijian Gong:** Visualization, Validation, Formal analysis. **Caiyun Wu:** Validation, Formal analysis, Data curation. **Wenbin Rong:** Validation, Formal analysis. **Ting Zhang:** Validation, Data curation, Conceptualization. **Qiaomei Li:** Validation, Formal analysis. **Hongjie Lei:** Writing – review & editing, Validation, Supervision, Funding acquisition, Data curation, Conceptualization.

## Declaration of competing interest

The authors state that they possess no recognized financial conflicts of interest or personal connections that might have seemed to impact the findings presented in this publication.

## Data Availability

The data produced and/or analyzed in the current study are available from the corresponding author on reasonable request.

## Appendix A Supplementary data

Supplementary material Image 1
