# Supplementary material for: Comparative evaluation of pure non-Saccharomyces yeasts fermentation and Lactiplantibacillus plantarum co-fermentation in fig pulp: Achieving remarkable sugar reduction and flavor enhancement
Source: Food Chem X. 2025 May 20;28:102570. doi: 10.1016/j.fochx.2025.102570 (PMC12152561; doi:10.1016/j.fochx.2025.102570)
Supplement: Supplementary file 1 — Supplementary material [file mmc1.docx]

Table S1 Alcohol content of fig pulp before and after fermentation (%, v/v).

|  | Control | TD | TD-Lp90 | LT | LT-Lp90 | MP | MP-Lp90 |
| --- | --- | --- | --- | --- | --- | --- | --- |
| Alcohol content | 0.23 ± 0.06^f^ | 3.70 ± 0.00^a^ | 3.27 ± 0.06^c^ | 3.30 ± 0.00^c^ | 3.20 ± 0.00^d^ | 1.57 ± 0.06^e^ | 3.50 ± 0.00^b^ |

Results are expressed as mean value of three determinations ± standard deviation. Values in the same row with different superscript letters are significantly different (*p* < 0.05).

Table S2 VOCs content in fig pulp before and after fermentation (mg/L).

|  | Volatile compounds | Control | TD | TD-Lp90 | LT | LT-Lp90 | MP | MP-Lp90 |
| --- | --- | --- | --- | --- | --- | --- | --- | --- |
| *Alcohols* |  |  |  |  |  |  |  |  |
| 1 | Ethanol | 42.09 | 187.97 | 261.37 | 246.85 | 256.89 | 143.46 | 189.89 |
| 2 | 3-Methyl-1-butanol | 101.42 | 103.82 | 90.45 | 116.29 | 108.45 | 110.80 | 93.93 |
| 3 | 1-Heptanol | 1.37 | ND | ND | ND | ND | 0.88 | ND |
| 4 | 1-Octen-3-ol | 2.40 | 1.62 | ND | ND | ND | 1.94 | ND |
| 5 | Phenethyl alcohol | 6.62 | 4.77 | 7.72 | 4.11 | ND | 9.94 | 14.72 |
| 6 | 1-Hexanol | 6.94 | ND | ND | ND | ND | ND | ND |
| 7 | (Z)-3-decenol | ND | ND | 0.28 | ND | ND | ND | 0.43 |
| 8 | 2,3-Butanediol | ND | ND | ND | ND | 3.24 | ND | ND |
| 9 | 1-Nonanol | ND | ND | ND | ND | ND | 0.45 | 0.29 |
| 10 | 2-Methyl-1-propanol | ND | ND | ND | ND | ND | 37.75 | ND |
| 11 | Trans-2-Octen-1-ol | ND | ND | ND | ND | ND | 0.41 | ND |
| Subtotal |  | 223.61 | 506.83 | 641.86 | 634.79 | 646.14 | 469.76 | 509.81 |
|  |  |  |  |  |  |  |  |  |
| *Esters* |  |  |  |  |  |  |  |  |
| 12 | 1-Methylhepty lacetate | 3.77 | ND | ND | ND | ND | 0.93 | ND |
| 13 | Ethyl acetate | 18.62 | 1.38 | ND | ND | ND | 12.11 | ND |
| 14 | Isoamyl acetate | 4.26 | ND | 2.17 | 2.47 | 4.52 | 3.10 | 4.79 |
| 15 | Ethyl caprylate | 1.61 | 13.19 | 16.34 | 8.83 | 19.30 | 0.69 | 13.86 |
| 16 | Ethyl lactate | ND | ND | 2.73 | ND | 2.11 | ND | 2.04 |
| 17 | Hexyl formate | ND | ND | 0.78 | 2.66 | 3.21 | 3.68 | 2.00 |
| 18 | Lactic acid isoamyl ester | ND | ND | 1.32 | 0.57 | 0.90 | ND | 1.51 |
| 19 | Ethyl pelanoate | ND | ND | 0.21 | ND | ND | ND | ND |
| 20 | Methyl decanoate | ND | 0.28 | 0.23 | 0.40 | 0.67 | ND | ND |
| 21 | Ethyl caprate | ND | 32.86 | 10.23 | 24.85 | 27.15 | ND | 9.06 |
| 22 | Ethyl laurate | ND | 1.62 | 1.04 | 0.79 | 2.20 | ND | 1.18 |
| 23 | Ethyl myristate | ND | 0.22 | 0.21 | ND | 0.38 | ND | 0.20 |
| 24 | Ethyl 9-hexadecenoate | ND | 0.15 | 0.18 | ND | 0.26 | ND | ND |
| 25 | Palmitic acid ethyl ester | ND | ND | 0.27 | ND | 0.33 | ND | 0.25 |
| 26 | Ethyl Hexanoate | ND | ND | ND | ND | 7.91 | ND | 4.44 |
| 27 | Diisobutyl phthalate | ND | ND | ND | ND | 0.22 | ND | ND |
| 28 | Ethyl heptanoate | ND | ND | ND | ND | ND | ND | 0.27 |
| 29 | Phenethyl acetate | ND | ND | ND | ND | ND | ND | 0.22 |
| Subtotal |  | 28.25 | 49.69 | 35.71 | 40.58 | 69.17 | 20.51 | 39.81 |
|  |  |  |  |  |  |  |  |  |
| *Aldehydes* |  |  |  |  |  |  |  |  |
| 30 | Acetaldehyde | 7.64 | ND | ND | ND | ND | ND | ND |
| 31 | Benzaldehyde | ND | 9.74 | 12.92 | 6.33 | 10.18 | ND | 13.42 |
| 32 | (E)-2-Octenal | ND | 0.43 | 0.74 | 0.63 | ND | ND | 1.31 |
| 33 | Hexanal | ND | ND | 1.57 | 2.10 | 1.93 | ND | ND |
| 34 | Phenylacetaldehyde | ND | 0.49 | ND | ND | ND | ND | ND |
| 35 | m-Tolualdehyde | ND | ND | ND | ND | 0.21 | ND | ND |
| 36 | Heptaldehyde | ND | ND | ND | 0.45 | ND | ND | 0.96 |
| 37 | 2-Methylbenzaldehyde | ND | ND | ND | ND | ND | ND | 0.39 |
| 38 | Trans-2-dodecenal | ND | ND | ND | ND | ND | ND | 0.24 |
| Subtotal |  | 7.64 | 10.66 | 15.22 | 9.5 | 12.33 | 0 | 16.34 |
|  |  |  |  |  |  |  |  |  |
| *Ketones* |  |  |  |  |  |  |  |  |
| 39 | 2-Octanone | 31.77 | 1.72 | 0.81 | 0.82 | 0.91 | 3.39 | 0.9 |
| 40 | Acetoin | 42.33 | ND | ND | ND | ND | ND | ND |
| 41 | 1-Octen-3-one | ND | ND | 1.16 | 1.03 | ND | ND | 1.99 |
| 42 | Damascenone | ND | ND | 14.86 | ND | ND | ND | 0.47 |
| Subtotal |  | 74.1 | 1.72 | 16.84 | 1.85 | 0.91 | 3.39 | 3.36 |
|  |  |  |  |  |  |  |  |  |
| *Terpenes* |  |  |  |  |  |  |  |  |
| 43 | (+)-Dipentene | 0.26 | ND | ND | 0.3 | ND | ND | 0.47 |
| 44 | Alpha-bulnesene | ND | ND | 0.5 | 0.41 | 0.37 | 0.41 | 0.69 |
| 45 | Styrene | ND | 0.95 | ND | ND | ND | ND | ND |
| Subtotal |  | 0.26 | 0.95 | 0.5 | 0.71 | 0.37 | 0.41 | 1.17 |
|  |  |  |  |  |  |  |  |  |
| *Acids* |  |  |  |  |  |  |  |  |
| 46 | Acetic acid | 6.07 | ND | ND | ND | ND | 3.77 | ND |
| 47 | Nonanoic acid | ND | ND | 1.52 | ND | ND | ND | ND |
| 48 | Octanoic acid | ND | 0.37 | 0.69 | 0.41 | 1.53 | ND | 0.85 |
| 49 | 4-Methylvaleric acid | ND | ND | ND | 1.85 | ND | ND | 2.02 |
| Subtotal |  | 6.07 | 0.37 | 2.22 | 2.26 | 1.53 | 3.77 | 2.87 |
| Total |  | 339.93 | 570.41 | 712.35 | 689.69 | 730.44 | 497.85 | 573.34 |

Results are the mean value of three independent replicates. Standard errors were always lower than 10% of mean value. ND, not detected.
